# Aislamiento y caracterización de una cepa temprana de SARS-CoV-2 durante la epidemia de 2020 en Medellín, Colombia

**DOI:** 10.7705/biomedica.5834

**Published:** 2020-11-15

**Authors:** Francisco J. Díaz, Wbeimar Aguilar-Jiménez, Lizdany Flórez-Álvarez, Gladys Valencia, Katherine Laiton-Donato, Carlos Franco-Muñoz, Diego Álvarez-Díaz, Marcela Mercado-Reyes, María T. Rugeles

**Affiliations:** 1 Grupo de Inmunovirología, Facultad de Medicina, Universidad de Antioquia, Medellín, Colombia Universidad de Antioquia Universidad de Antioquia Medellín Colombia; 2 Ayudas Diagnósticas de Laboratorio Clínico, ADILAB, Medellín, Colombia ADILAB Medellín Colombia; 3 Unidad de Secuenciación Genómica, Instituto Nacional de Salud, Bogotá, D.C., Colombia Instituto Nacional de Salud BogotáD.C Colombia

**Keywords:** infecciones por coronavirus, síndrome respiratorio agudo grave, virus del SARS, secuenciación de nucleótidos de alto rendimiento, microscopia electrónica, técnica indirecta del anticuerpo fluorescente, Coronavirus infections, viral isolation severe acute respiratory syndrome, SARS virus, high-throughput nucleotide sequencing, microscopy, electron, fluorescent antibody technique, indirect

## Abstract

**Introducción.:**

El nuevo coronavirus causante de un brote de enfermedad respiratoria aguda en China en diciembre de 2019 se identificó como SARS-CoV-2. La enfermedad, denominada COVID-19, fue declarada pandemia por la Organización Mundial de la Salud (OMS). El primer caso de COVID-19 en Colombia se reportó el 6 de marzo de 2020; en este estudio se caracterizó un aislamiento temprano del virus SARS-CoV-2 de una muestra recolectada en abril de 2020.

**Objetivos.:**

Describir y caracterizar una cepa temprana a partir de un aislamiento de SARS-CoV-2 durante la pandemia en Colombia.

**Materiales y métodos.:**

Se obtuvo una muestra de un paciente con COVID-19 confirmada por qRT-PCR; la muestra fue inoculada en diferentes líneas celulares hasta la aparición del efecto citopático. Para confirmar la presencia de SARS-CoV-2 en el cultivo, se utilizó la qRT-PCR a partir de los sobrenadantes, la inmunofluorescencia indirecta (IFI) en células Vero-E6, así como microscopía electrónica y secuenciación de nueva generación *(next-generation sequencing).*

**Resultados.:**

Se confirmó el aislamiento de SARS-CoV-2 en células Vero-E6 por la aparición del efecto citopático tres días después de la infección, así como mediante la qRT-PCR y la IFI positiva con suero de paciente convaleciente positivo para SARS-CoV-2. Además, en las imágenes de microscopía electrónica de trasmisión y de barrido de células infectadas se observaron estructuras compatibles con viriones de SARS-CoV-2. Por último, se obtuvo la secuencia completa del genoma, lo que permitió clasificar el aislamiento como linaje B.1.5.

**Conclusiones.:**

La evidencia presentada en este artículo permite confirmar el primer aislamiento de SARS-CoV-2 en Colombia. Además, muestra que esta cepa se comporta en cultivo celular de manera similar a lo reportado en la literatura para otros aislamientos y que su composición genética está acorde con la variante predominante en el mundo. Finalmente, se resalta la importancia que tiene el aislamiento viral para la detección de anticuerpos, para la caracterización genotípica y fenotípica de la cepa y para probar compuestos con potencial antiviral.

## Abstract (en)

**Introduction:**

SARS-CoV-2 has been identified as the new coronavirus causing an outbreak of acute respiratory disease in China in December, 2019. This disease, currently named COVID-19, has been declared as a pandemic by the World Health Organization (WHO). The first case of COVID-19 in Colombia was reported on March 6, 2020. Here we characterize an early SARS-CoV-2 isolate from the pandemic recovered in April, 2020.

**Objective:**

To describe the isolation and characterization of an early SARS-CoV-2 isolate from the epidemic in Colombia.

**Materials and methods:**

A nasopharyngeal specimen from a COVID-19 positive patient was inoculated on different cell lines. To confirm the presence of SARS-CoV-2 on cultures we used qRT-PCR, indirect immunofluorescence assay, transmission and scanning electron microscopy, and next-generation sequencing.

**Results:**

We determined the isolation of SARS-CoV-2 in Vero-E6 cells by the appearance of the cytopathic effect three days post-infection and confirmed it by the positive results in the qRT-PCR and the immunofluorescence with convalescent serum. Transmission and scanning electron microscopy images obtained from infected cells showed the presence of structures compatible with SARS-CoV-2. Finally, a complete genome sequence obtained by next-generation sequencing allowed classifying the isolate as B.1.5 lineage.

**Conclusion:**

The evidence presented in this article confirms the first isolation of SARS-CoV-2 in Colombia. In addition, it shows that this strain behaves in cell culture in a similar way to that reported in the literature for other isolates and that its genetic composition is consistent with the predominant variant in the world. Finally, points out the importance of viral isolation for the detection of neutralizing antibodies, for the genotypic and phenotypic characterization of the strain and for testing compounds with antiviral potential.

En diciembre de 2019 en Wuhan, República Popular de China, se describieron los primeros casos de una enfermedad febril con neumonía grave que se presentaba con un espectro clínico variable y, en algunos casos, resultados fatales, principalmente en adultos mayores y en pacientes con ciertas comorbilidades ^1^^,^^2^. A principios de 2020 se aisló en muestras de lavado broncoalveolar un nuevo agente que se identificó como un betacoronavirus ^2^^,^^3^. Este virus, inicialmente denominado 2019-nCoV *(novel coronavirus 2019),* se conoce hoy como SARS-CoV-2 por su similitud genética y clínica con otro betacoronavirus, el SARS-CoV, causante de la epidemia de síndrome respiratorio agudo grave *(Severe Acute Respiratory Syndrome,* SARS) que se presentó entre 2002 y 2003 en Asia y que se diseminó de forma limitada a otros países ^4^.

La enfermedad causada por el SARS-CoV-2, ahora denominada COVID-19 *(Coronavirus Disease 2019),* se ha extendido por todo el mundo causando más de 25 millones de casos confirmados y más de 850.000 muertes en los primeros ocho meses, lo que la ha convertido en la mayor pandemia de los últimos tiempos *(Coronovairus resource center, Johns Hopkins University (JHU) Global,*https://coronavirus.jhu.edu/map.html).

El origen de los coronavirus que afectan a los humanos parece ser zoonótico; de hecho, los betacoronavirus humanos están filogenéticamente relacionados con los coronavirus de murciélagos y pueden transmitirse a los humanos directamente o por medio de un huésped intermediario; según se ha sugerido, este es el caso del SARS-CoV-2 ^4^^,^^5^.

Al ser un virus con genoma ARN, el SARS-CoV-2 tiene una alta tasa de evolución, lo que ha llevado a que durante los pocos meses de la pandemia se haya descrito un gran número de variables ^6^. Entre los cambios genéticos ocurridos en los primeros meses de 2020 se destaca la mutación D614G, un cambio de ácido aspártico por glicina en la proteína S *(spike)* que hace más eficiente la unión del virus a su receptor en células humanas y que hoy predomina en todos los continentes ^7^. El efecto de estas variantes en términos de complicaciones clínicas se desconoce, aunque se ha establecido una correlación positiva entre la variante G614 y la tasa de mortalidad ^8^.

El primer caso confirmado de COVID-19 en Colombia se reportó el 6 de marzo de 2020 ^9^. En este estudio se describe el primer aislamiento del virus SARS-CoV-2 en Colombia -en abril de 2020- a partir de una muestra de aspirado nasofaríngeo de un paciente de Medellín.

## Descripción del caso

El aislamiento provino de una muestra de aspirado nasofaríngeo de uno de los primeros pacientes diagnosticados con COVID-19 en Colombia.

Se trataba de un hombre de 59 años residente en Medellín con antecedentes de hipertensión arterial, diabetes mellitus e hipercolesterolemia, todas ellas en tratamiento en el momento de la infección.

El paciente había viajado por el norte de España, Madrid y Portugal durante 17 días y regresó a Medellín el 12 de marzo de 2020. A su llegada se le ordenó permanecer en confinamiento domiciliario. Cuatro días más tarde, el 16 de marzo, tuvo los primeros síntomas que incluían cefalea, dolor dorsal alto, malestar en la faringe, tos leve y fiebre (38,2 °C). En los días siguientes, el dolor dorsal se extendió por la espalda y el paciente presentó somnolencia, pérdida del apetito y ageusia sin anosmia, y se le trató únicamente con acetaminofén.

El 19 de marzo se le tomó un aspirado nasofaríngeo, el cual resultó positivo en la prueba de qRT-PCR (protocolo del Charité, Berlín) ^10^. En los días siguientes a la toma de la muestra el paciente presentó astenia prolongada y malestar mal definido en la espalda, pero con tendencia a la mejoría. El 25 de marzo, aún con sintomatología leve, se tomó otra muestra de aspirado nasofaríngeo y una de sangre para el aislamiento viral y la determinación de los títulos de anticuerpos, respectivamente. El 14 de abril (después del aislamiento) se tomó una nueva muestra de sangre durante la fase convaleciente. En el último control clínico, realizado por teléfono el 19 de abril, el paciente informó estar completamente recuperado.

## Materiales y métodos

### Muestras

Las muestras de sangre y aspirado nasofaríngeo se tomaron en las fechas mencionadas. El paciente dio su consentimiento después de ser informado sobre el propósito de la investigación. Parte de la muestra del aspirado nasofaríngeo fue sometida a la prueba de qRT-PCR para SARS-CoV-2 y el resto se conservó a -80 °C para el aislamiento viral.

### Aislamiento viral

El aislamiento viral se hizo en el laboratorio de nivel 3 de bioseguridad (BSL-3) de la Sede de Investigación Universitaria de la Universidad de Antioquia, siguiendo las prácticas y procedimientos recomendados ^11^.

Se emplearon las líneas celulares LLC-MK2, Vero-76 y Vero-E6. Las células se cultivaron en DMEM *(Dulbecco's Modif'ed Eagle Médium,* Sigma-Aldrich, St. Louis, MO, USA) con suplemento de suero bovino fetal (SBF) (Gibco, Grand Island, NY, USA) al 10 % y penicilina-estreptomicina (Sigma-Aldrich, St. Louis, MO, USA) al 1 % en frascos de cultivo celular de 25 cm^2^. Una vez alcanzada una confluencia del 80 % se procedió a inocular la monocapa con 80 µl del aspirado nasofaríngeo diluidos en 1 ml de medio DMEM. Las células se incubaron a 37 °C con 5 % de CO_2_ durante 90 minutos agitando suavemente cada 15 minutos. Después de la incubación se retiró el inóculo y se reemplazó con 5 ml de medio DMEM con 2 % de SBF y 1 % de penicilina-estreptomicina. Los cultivos se inspeccionaron bajo el microscopio diariamente para detectar el efecto citopático. Para cada línea celular se incluyó un control sin infección para determinar la apariencia de las células en ausencia del efecto citopático.

### Detección del SARS-CoV-2 mediante RT-PCR en tiempo real

Se extrajo ARN viral a partir de la muestra de aspirado nasofaríngeo del paciente y de los sobrenadantes del cultivo de células inoculadas usando el estuche comercial QlAamp Viral RNA Mini Kit™ (Qiagen, Hilden, Germany) según las instrucciones de la casa comercial.

La transcripción inversa y la posterior amplificación del genoma viral del SARS-CoV-2 en tiempo real (qRT-PCR) se hizo en un solo paso utilizando el estuche comercial qScript XLT 1-Step RT-qPCR Tough Mix™ (Quantabio Beverly, MA, USA) con los oligonucleótidos y sondas del protocolo CDC RT-PCR para el gen *N1* (IDT, Coralville, lowa USA) (secuencias disponibles en https://www.cdc.gov/coronavirus/2019-ncov/lab/rt-pcr-panel-primer-probes.html).

### Inmunofluorescencia indirecta

Para confirmar el aislamiento viral se utilizó inmunofluorescencia indirecta (IFI) en placas sensibilizadas con células Vero-E6. A los cuatro días de cultivo se desprendieron mecánicamente las monocapas de células Vero-E6 inoculadas y no inoculadas y se suspendieron de nuevo en 3 ml de tampón fosfato salino (PBS) (Lonza, Rockland, ME, USA). Se agregaron 20 µl/pozo de la suspensión de células en láminas portaobjetos de 12 pozos (Thermo Scientific, Wilmington, DE, USA), se dejaron secar y luego se fijaron por inmersión en acetona pura durante 15 minutos.

A partir del suero del paciente se hicieron diluciones seriadas dobles desde 1:5 hasta 1:80 en PBS. Se agregaron 20 µl de cada dilución a las células fijadas en las láminas y se incubaron en cámara húmeda a 37 °C durante 30 minutos. Después de la incubación las placas se lavaron dos veces con PBS durante 5 minutos con agitación lenta, se dejaron secar y en cada pozo se agregaron 20 µl de conjugado Anti-human IgG (Fc specific)-FITC antibody produced in goat™ (Sigma-Aldrich) en PBS. Las placas se incubaron en cámara húmeda a 37 °C durante 30 minutos protegidas de la luz, luego se lavaron dos veces con PBS y se montaron con anti-fade Fluosaver™ (Calbiochem) y lámina cubreobjetos. Las placas se visualizaron en un microscopio invertido de luz fluorescente Axio Vert.A1™ (Zeiss, Oberkochen, Alemania) con 400X.

### *Caracterización viral mediante microscop*í*a electrónica*

El estudio ultraestructural del virus se hizo mediante microscopía electrónica de transmisión y microscopía electrónica de barrido en el Centro de Microscopía Avanzada de la Universidad de Antioquia. Para la microscopía electrónica de transmisión se procesaron células Vero-E6 inoculadas con la muestra nasofaríngea después de la aparición del efecto citopático. Estas se fijaron con glutaraldehído al 2,5 % y posteriormente se fijaron con tetróxido de osmio al 1-2 %. A continuación, las células se lavaron en PBS dos veces y se trataron con alcoholes en concentraciones ascendentes (70, 95 y 100 %) para deshidratarlas. Después, las muestras se embebieron en resina epóxica y se sometieron a polimerización en cápsulas para obtener bloques de los cuales se sacaron cortes de 60 a 90 nanómetros con un ultramicrótomo. Estos se montaron en rejillas y se contrastaron inicialmente con acetato de uranilo y luego con citrato de plomo. Los cortes en las rejillas se observaron en un microscopio electrónico de transmisión Tecnai G2 F20™ (FEI Company, Hillsboro, OR, USA).

Para la microscopía electrónica de barrido las muestras deshidratadas se colocaron en un secador de punto crítico SPC SAMDRI-795™ (Tousimis, Rockville, MD, USA). Luego se fijaron en una cinta de grafito, se les realizó un recubrimiento delgado en oro y se analizaron en el microscopio electrónico de barrido JEOL JSM 6490 LV™ (JEOL; Peabody, MA, USA) en alto vacío. Se empleó el detector de electrones secundarios para evaluar la morfología y la topografía de las muestras.

### Cuantificación de los títulos virales mediante ensayo en placa

El virus aislado se tituló mediante ensayo en placa de monocapas de células Vero-E6. Las células se cultivaron en medio DMEM con suplemento de 10 % de SBF a 37 °C y 5 % de CO_2_. Para preparar las placas se sembraron células Vero-E6 en una densidad de 1 x 10^5^ células/pozo en platos de 24 pozos con 500 µl de medio DMEM y suplemento de 2 % de SBF; las células se incubaron durante 24 horas a 37 °C con 5 % de CO_2_ y después de la incubación se infectaron durante una hora con 200 µl/pozo de diluciones en base diez del aislamiento viral a 37 °C con 5 % de CO_2._ Pasada una hora, se retiró el inóculo y se reemplazó con 1 ml de medio semisólido (DMEM con 2 % de SFB más 1,5 % de carboximetilcelulosa). Las células se incubaron durante cuatro días a 37 °C con 5 % de CO_2._

Pasados cuatro días se retiró el medio semisólido y se lavaron las células dos veces con PBS. Posteriormente, se hicieron la tinción y fijación con 500 µl por pozo de una solución de formaldehído al 4 % y cristal violeta al 1 % durante 30 minutos a temperatura ambiente. Por último, las células se lavaron dos veces con PBS.

Para determinar el título viral, se hizo el recuento de las placas. El promedio de dos réplicas se multiplicó por el inverso de la dilución y el volumen del inóculo para obtener el número de unidades formadoras de placa por ml (UFP/ml). Para establecer el título del aislamiento en UFP/ml se hicieron tres experimentos independientes.

### Secuenciación de nueva generación

Para secuenciar el genoma se utilizó el ARN extraído del sobrenadante del cultivo de células con efecto citopático y resultado positivo en la qRT-PCR. La preparación y secuenciación de la librería se hizo con tecnologías de nanopore siguiendo el protocolo ARTIC ^12^. Se obtuvo un conjunto de amplicones de ~400 pb de todo el genoma del SARS-CoV-2 con los cebadores nCoV-2019/V3 ^13^.

Los amplicones se mezclaron, se cuantificaron y se etiquetaron con el estuche Native Barcoding Kit EXPNBD104™ (Oxford Nanopore Technologies, Oxford, UK) y se combinaron en una cantidad equimolar. Se prepararon librerías genómicas con el estuche de ligadura 1D SQK-LSK109™ (Oxford Nanopore Technologies) y se secuenciaron usando una celda de flujo FLO-MIN106-R9.4™ y el instrumento MinION™ (Oxford Nanopore Technologies).

Las bases nitrogenadas se identificaron usando Guppy, versión 3.2.2™ (Oxford Nanopore Technologies). Las lecturas procesadas se alinearon con el genoma de referencia del SARS-CoV-2 (GenBank NC_045512.2) utilizando el algoritmo BWA-MEM ^14^ y el BBMap (https://www.osti.gov/biblio/1241166-bbmap-fast-accurate-splice-aware-aligner) para generar la secuencia de consenso. Por último, el linaje de la secuencia se clasificó usando PANGOLIN (Phylogenetic Assignment of Named Global Outbreak LINeages) ^15^.

## Resultados

### Aislamiento viral

**Figura 1:**
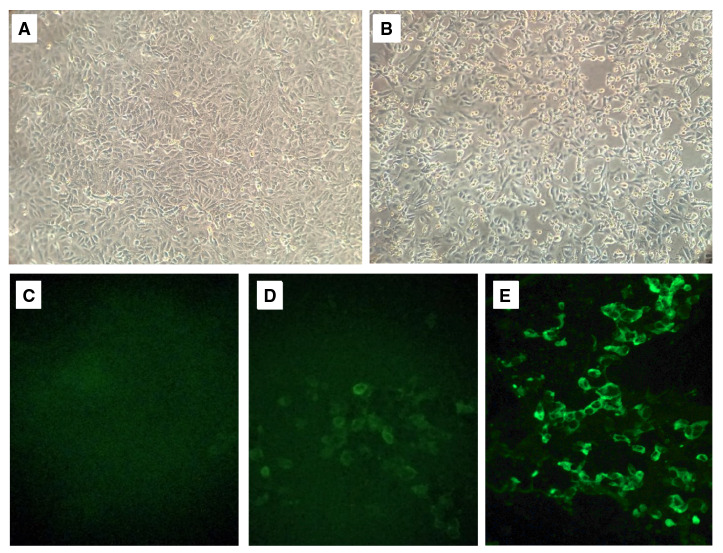
Identificación de un aislamiento colombiano de SARS-CoV-2. A. y B. Monocapa de células Vero-E6 sin infectar e infectadas; se observa el efecto citopático del SARS-CoV-2 en monocapas de células Vero-E6 a tres días de la inoculación. C., D. y E. Fotografías de placas de IFI preparadas con el suero del paciente en células no infectadas, suero de la etapa aguda en células infectadas y suero de convaleciente en células infectadas

Antes del aislamiento se hizo la qRT-PCR a la muestra tomada el 25 de marzo, la cual resultó positiva con un ciclo umbral de 19, lo que demostró que el paciente seguía excretando gran cantidad de virus. A los tres días de la infección, las monocapas de células control eran confluentes, en tanto que las monocapas inoculadas con la muestra mostraban un efecto citopático significativo (figura 1A-B). Se tomaron muestras de sobrenadante y se incubó el cultivo hasta el día siguiente. Al cuarto día de la infección se observó un desprendimiento del 65 % de la monocapa y se recolectaron las células para la IFI.

Con el sobrenadante recolectado a los tres días de la infección se hizo la qRT-PCR con oligos específicos para el gen *N* del SARS-CoV-2, con resultado positivo y un ciclo umbral de 12 ciclos. En la IFI realizada con el primer suero del paciente se observó una fluorescencia de baja intensidad (figura 1 C y D). Sin embargo, cuando se repitió con el suero del paciente ya convaleciente se obtuvo una fluorescencia con un patrón periférico bien definido (figura 1E).

### Caracterización viral mediante microscopia electrónica

**Figura 2:**
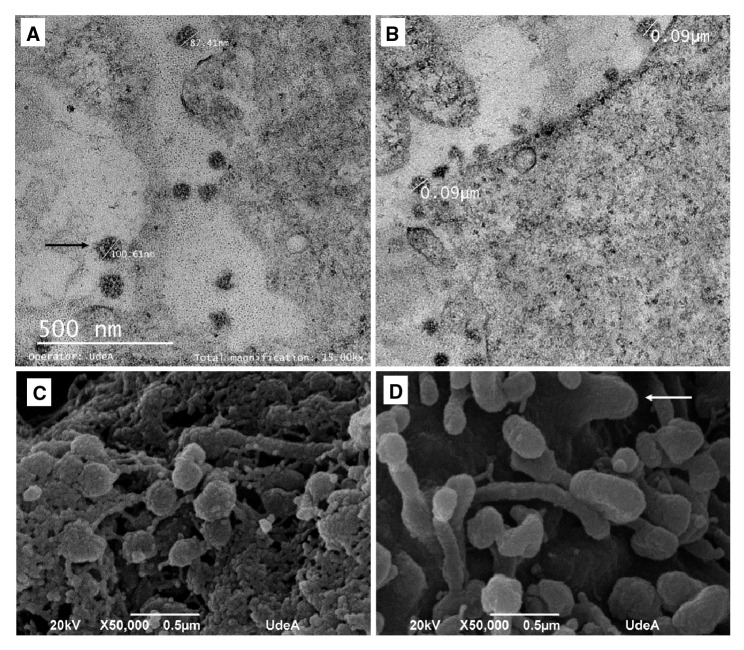
Microscopía electrónica de células Vero-E6 infectadas. A. y B. Fotografías obtenidas por microscopía electrónica de transmisión de partículas virales con tamaño variable entre 87 y 180 nm de diámetro; en A. la flecha señala una estructura compatible con espículas de viriones de SARS-CoV-2. C. y D. Fotografías obtenidas por microscopía electrónica de barrido; se observan estructuras con morfología y tamaño compatibles con de viriones de SARS-CoV-2. En D. también se observa un virión en proceso de gemación (flecha).

Para comprender mejor la morfología del aislamiento de SARS-CoV-2 se utilizó microscopía electrónica en las partículas virales. En la muestra de células Vero-E6 con efecto citopático observadas con microscopía electrónica de transmisión, estas presentaron tamaños entre los 86 y los 180 nm de diámetro (figura 2A y 2B). Aunque no se evidenció en ellas la típica apariencia de corona, sí se observaron algunas estructuras compatibles con espículas (figura 2A). En cortes finos las espículas virales no suelen preservarse (Dr. Vsevolod Popov, UTMB, Galveston TX, USA, comunicación personal). La forma y el tamaño de los viriones se confirmaron mediante microscopía electrónica de barrido. Las partículas virales se observan redondas y llenas, algunas de ellas adheridas unas a otras (figura 2C). También se observaron imágenes sugestivas de viriones en proceso de gemación y partículas elongadas de identidad desconocida (figura 2D).

### Cuantificación de los títulos virales mediante ensayo en placa

**Figura 3:**
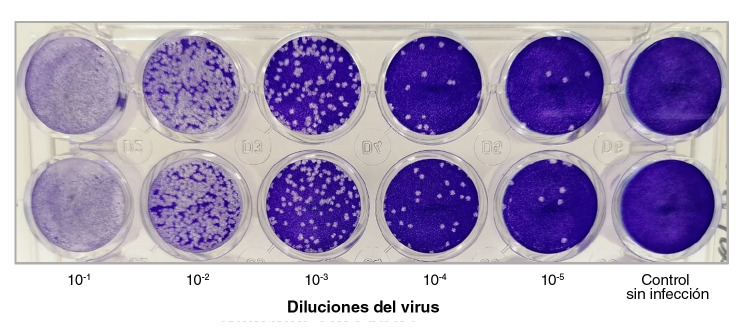
Placas con SARS-CoV-2 en monocapa de células Vero-E6. Esta imagen es representativa de la preparación de placas con SARS-CoV-2 en monocapas de células Vero-E6. Se fijaron y se hizo su tinción con una solución de 4 % de formaldehído y 1 % de cristal violeta a los cuatro días de la infección.

Se obtuvieron títulos de 3,4 ± 0,7 x 10^6^ UFP/ml a los 4 días después de la infección en el ensayo en placas con células Vero E6. En estas monocapas se evidenció que el SARS-CoV-2 generó dos tamaños de placa como se observa en la figura 3.

### Caracterización genómica del aislamiento viral

El análisis de la secuencia genómica obtenida evidenció una profundidad de 4.600 x o más y una cobertura del 99,5 %. Cuando se comparó con la secuencia de referencia NC_045512.2 (Wuhan-HU1-2019), se observaron cuatro diferencias en las posiciones 241, 3.037, 14.408 y 23.403 del genoma. Estas dos últimas son sustituciones no sinónimas y corresponden a las mutaciones P323L en la polimerasa viral y D614G en la proteína S del SARS-CoV-2. El genoma se clasificó como linaje B.1.5 con un valor de *bootstrap* de 92 y un valor de SH-aLTR de 100, resultado que coincide con linajes previamente descritos en el país ^16^. La secuencia fue depositada en la base de datos GISAID (www.gisaid.org) con el nombre hCoV-19/Colombia/ANT-UdeA-200325-01/2020 y código de acceso EPI ISL 536399.

## Discusión

El objetivo de este estudio era aislar una cepa de SARS-CoV-2 a partir de una muestra de un paciente con COVID-19. Los resultados obtenidos mediante las pruebas qRT-PCR e IFI, y las imágenes de microscopía electrónica de transmisión y de barrido y la secuenciación de nueva generación en el cultivo celular inoculado con la muestra nasofaríngea del paciente estudiado permitieron concluir más allá de cualquier duda que se logró aislar una cepa de SARS-CoV-2.

Este es el primer aislamiento del virus en Colombia. Se evidenció que la cepa se comportó *in vitro* de manera similar a lo descrito para otras cepas de SARS-CoV-2: el efecto citopático apareció en el tercer día con encogimiento y desprendimiento celular y un patrón mixto de placas grandes y pequeñas en el ensayo en placas con **c**élulas Vero-E6 ^17^^,^^18^.

El aislamiento se logró a pesar de que la muestra fue tomada menos de 13 días después de la infección y a nueve días del inicio de los síntomas. Se observó un ciclo umbral de 19 en la rRT-PCR y se alcanzó un crecimiento viral solo tres días después de la inoculación del cultivo. Esto indica una replicación eficiente del virus, con una alta excreción de partículas virales infecciosas, por lo menos, en algunos pacientes sintomáticos con COVID-19. Este resultado alerta sobre la inconveniencia de reducir el período de aislamiento de los pacientes infectados, que originalmente era de 14 días pero luego se redujo a diez ^19^.

La secuenciación de nueva generación permitió la identificación de cuatro sustituciones en las posiciones 241, 3.037, 14.408 y 23.403 del genoma viral y del linaje genético B.1.5" La última de dichas mutaciones representa la sustitución D614G a nivel de aminoácidos en la proteína S o espícula del virión. Dicha proteína incluye el dominio de unión al receptor y, por consiguiente, determina el tropismo tisular del virus; además, constituye el principal antígeno viral, dado que los anticuerpos neutralizantes se unen específicamente a dicha proteína.

En ensayos con pseudovirus se ha demostrado que las variantes G614 generan títulos virales mayores comparados con las D614 en diferentes líneas celulares ^7^. Esta mayor capacidad infecciosa se ha correlacionado con un aumento de la estabilidad y la tasa de incorporación de la proteína S en la membrana de los viriones ^20^. Se ha demostrado, además, que la mutación D614G aumenta la capacidad replicativa *in vitro* del SARS-CoV-2 en células epiteliales de pulmón y en cultivos primarios de tejido de las vías respiratorias ^21^. Aunque en modelos animales no se observaron diferencias significativas en los signos clínicos como la pérdida de peso, sí se ha reportado que las variantes G614 producen mayores cargas virales en los lavados traqueales y nasales, pero no en los pulmonares ^21^. Por otra parte, se ha reportado que la infección con variantes D614 genera una mejor actividad de anticuerpos neutralizantes contra G614, lo que sugiere que esta mutación no reduciría la inmunidad conferida por la primera de estas variantes en infectados o vacunados contra la COVID-19 ^21^.

Con base en esta información se puede concluir que, aunque no hay evidencia de que las variantes G614 sean más letales, sí es cierto que la transmisión es más eficiente, lo que implica una ventaja evolutiva que podría explicar la rápida expansión de esta mutación. En efecto, los virus con la sustitución D614G incrementaron su frecuencia durante los primeros meses de la pandemia de COVID-19, incluso en regiones donde la D614 era dominante en un comienzo; esta transición se ha dado de manera asincrónica y en diferentes regiones alrededor del mundo: primero en Europa y Norteamérica y luego en Asia y Oceanía ^7^.

Como método diagnóstico, el aislamiento en cultivo celular se ha ido reemplazando por métodos más sensibles como la amplificación de genomas virales, incluida la prueba de qRT-PCR, que constituye el estándar diagnóstico para la infección por SARS-CoV-2; otras ventajas de los métodos moleculares son un menor tiempo de ejecución y un menor riesgo biológico para el laboratorista.

Sin embargo, en este estudio se pudo comprobar la utilidad del aislamiento viral para varios fines específicos: primero, para determinar el estado contagioso del paciente infectado; se ha observado que las muestras positivas para SARS-CoV-2 en la qRT-PCR con ciclos umbrales altos no se asocian con una excreción viable de virus, es decir, la qRT-PCR no permite diferenciar entre pacientes infectados y pacientes infecciosos ^22^.

Segundo, la disponibilidad del virus vivo permite la producción de antígenos virales utilizables en las pruebas de detección de anticuerpos por inmunofluorescencia indirecta (figuras 1D y 1E); además, dada la nítida formación de dichas placas en monocapas de células Vero-E6, la detección de anticuerpos neutralizantes con el método de reducción de placas cobrará cada vez más importancia a medida que se introduzcan las esperadas vacunas contra la COVID-19 (figura 3).

En tercer lugar, el aislamiento viral facilita la secuenciación del ARN viral, como lo demostró la cobertura del 99,5 % y la gran profundidad del genoma del virus aislado; a menudo la secuenciación directa a partir de muestras no permite obtener el genoma completo, especialmente en aquellas con baja carga viral.

Por último, la disponibilidad del virus vivo permite probar sustancias con posible acción antiviral, incluidos nuevos fármacos antivirales, antivirales de segundo uso, productos naturales y compuestos químicos o procesos físicos de desinfección para la limpieza de superficies, objetos o sustancias que puedan albergar virus infecciosos, procesos que hoy se están haciendo en nuestros laboratorios.
